# The use of a 3D-printed prosthesis in a Great Hornbill (*Buceros bicornis*) with squamous cell carcinoma of the casque

**DOI:** 10.1371/journal.pone.0220922

**Published:** 2019-08-13

**Authors:** Shangzhe Xie, Bohong Cai, Ellen Rasidi, Ching-Chiuan Yen, Chia-da Hsu, Wai Tung Chow, Virginie De Busscher, Li Chieh Hsu

**Affiliations:** 1 Wildlife Reserves Singapore, Singapore, Singapore; 2 Keio-NUS CUTE Center, Smart Systems Institute, National University of Singapore, Singapore, Singapore; 3 Division of Industrial Design, School of Design and Environment, National University of Singapore, Singapore, Singapore; 4 Veterinary Emergency & Specialty Hospital, Singapore, Singapore; 5 The Animal Clinic, Singapore, Singapore; Thomas Jefferson University, UNITED STATES

## Abstract

The advent of new technologies in medical imaging and 3D printing in recent years has made customization of surgical tools and implants more accessible, revolutionizing many surgical fields. In many human diseases, these implants have led to superior surgical outcomes and greatly improved patients’ quality of life. Thus, it is of great interest to apply these technologies to the treatment of animal diseases. In this study, we report the use of computed tomography (CT) and 3D printing for the treatment of a Great Hornbill at Jurong Bird Park that was diagnosed with squamous cell carcinoma of the casque. A 3D printed prosthesis that perfectly fitted the subject was implanted to replace its resected casque. The subject exhibited natural eating behaviour with no post-operative complications. Using this case as an example, the positive outcomes suggest a great potential in applying these technologies to the treatment of other wildlife diseases.

## Introduction

In recent years, combining medical imaging and 3D printing to create customized surgical guides and prostheses for human patients has become more common for a range of diseases [1–3]. Multi-slice advanced medical imaging data, particularly computed tomography (CT) and magnetic resonance imaging (MRI), can accurately reflect anatomical details of the scanned region and be converted to 3D volumetric models [4]. Based on these patient-specific 3D models, customized surgical tools can be created and perfectly fitted to the anatomy, allowing improved surgical planning that translates to superior surgical outcomes [5–7].

Similarly, customized surgical tools and implants have great potential for use in veterinary medicine to improve surgical efficiency and post-operative recovery [8,9]. However, unlike in humans, the specific features and natural behaviours of animals have to be considered when designing customized components for them. Many species face challenges not only in the post-operative recovery period, but also in adapting to and incorporating the prosthesis into normal behaviours necessary for survival and reproduction.

### Casque squamous cell carcinoma in Great Hornbills (*Buceros bicornis*)

The large casque on the dorsal maxillary beak, composed of pneumatized cancellous maxillary bone covered by the keratinous sheath of the rhamphotheca, is the hallmark of the great hornbill (*Buceros bicornis*) [10–12]. Squamous cell carcinoma of the casque is regularly reported in this hornbill species, and medical treatment is usually unrewarding [11,13]. As complete excision was the only treatment significantly associated with complete or partial response and increased survival in a retrospective study of squamous cell carcinomas in birds [14], resection of affected tissue including the casque rhinotheca and underlying maxillary bone was the preferred treatment option in this case. However, planning and performing radical resection accurately, and preventing post-operative complications is a challenge.

In the present study, a 22-year-old reproductively entire male great hornbill, held in a zoological collection (Jurong Bird Park, Singapore), was diagnosed with squamous cell carcinoma of the rostral maxillary beak and casque, thus requiring radical resection to remove the neoplastic tissue. The aim of this study was to combine medical imaging and 3D printing to design and produce a customized surgical guide and prosthesis to enable accurate radical resection of the required amount of tissue, whilst preserving the natural behaviour of the patient post-operatively.

## Methods

### Diagnosis and initial treatment

The patient was presented and admitted to the avian hospital at Jurong Bird Park for evaluation of a lesion on the rostral aspect of the casque. The admission weight was 2.34 kg and the patient appeared to be in good body and plumage condition. The following day, a full physical examination was performed under brief inhalation anaesthetic (preoxygenation with 100% oxygen via facemask, followed by isofluorane at 2% for induction and maintenance). Head and skull radiographs, as well as phlebotomy for complete blood count (CBC) and serum biochemistry, were performed. Increased heterogenous radio-opacity in the rostral area of the casque suggested an inflammatory or neoplastic process. Intramuscular injections of ceftiofur (Excede, ceftiofur 200 mg/ml, Zoetis, Rhodes, Australia), a broad-spectrum antibiotic, was prescribed at 20mg/kg every 72 hours. The only abnormality on bloodwork was a mild elevation in creatine kinase (CK), using the species global references ranges provided by ZIMS/Species360.

Seven days after initial presentation, the bird was sedated with a combination of midazolam (Midazolam-hameln injection, midazolam 5 mg/ml, Hameln Pharmaceuticals Ltd, Gloucester, United Kingdom) 0.2 mg/kg and butorphanol (Butomidor injection, butorphanol 10 mg/ml, Richter Pharma Ag, Wets, Austria) 0.5 mg/kg IM and transported to a local veterinary specialist centre (Veterinary Emergency and Specialty Hospital, Singapore) for a CT scan and CT-guided biopsy of the rostral casque lesion. The patient was preoxygenated with 100% oxygen delivered via face mask for five minutes under manual restraint and then induced with isofluorane at a concentration of 3%. The patient was then intubated with an uncuffed endotracheal tube and a light surgical plane of anaesthesia was maintained using 2% isofluorane in oxygen throughout the procedure. A 22G IV catheter was placed in the left ulnar vein and intravenous compound sodium lactate solution (Braun Medical Industries, Penang, Malaysia) administered at a rate of 10 ml/kg/h throughout the procedure.

The CT scan was performed using a 32-slice multi-detector CT unit (Revolution ACT, GE Healthcare). Images of the entire head and coelomic cavity were acquired as a volume with 1s rotation speed, 512 x 512 matrix, 120 KV, 100 mA, small field of view and slice thickness of 1.25 and 0.625 mm, reconstructed with soft tissue, pulmonary and bone algorithms. Images were reviewed using soft tissue, pulmonary and bone window settings. Nonionic iodinated contrast medium (Omnipaque 300, Iohexol 300 mg/ml, GE Healthcare, Oslo, Norway) at a dose of 2 ml/kg, was administered manually through the intravenous catheter.

**Fig 1:**
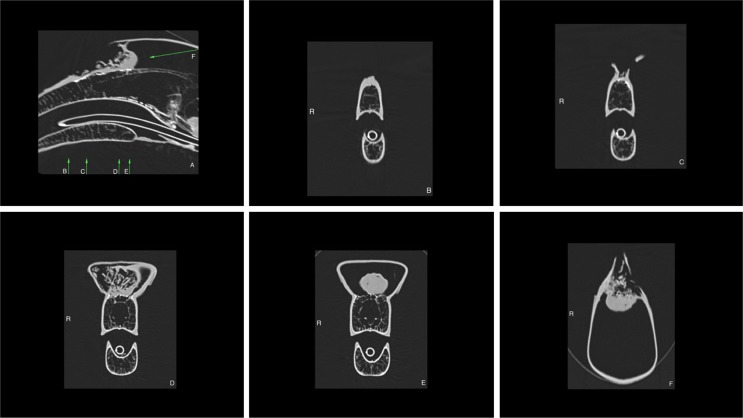
CT scan images of the head reconstructed using pulmonary algorithm showing the extent of the lesion in the casque. A: Sagittal image showing the mass, the destruction of the casque and the irregular soft tissue band extending up to the rostrodorsal aspect of the beak (the arrows indicate the plane sections of the images B, C, D, E and F); B: Transverse image at the level of the irregular band of abnormal soft tissue located dorsal to the beak (R: Right); C: Transverse image showing the destruction of the casque; D: Transverse image showing the amorphous appearance of the mass at its rostral aspect and the thickening of the nasal turbinates at the right ventral aspect of the mass; E: Transverse image showing the well-defined appearance of the mass at its caudal apsect; F: Dorsal image of the mass and casque.

The scan revealed a mildly contrast enhancing and aggressive mass at the rostroventral aspect of the casque. The mass appeared well-defined, compact, round and 30 mm in length, 27.5 mm in height, and 30 mm in width at its caudal aspect and ill-defined and irregular to amorphous at its rostral aspect where destruction of the casque was observed. Some fluid was seen around the rostral part of the mass. The mass extended rostrally as an irregular band of soft tissue of 6 mm to 2 mm in height and 10 mm to 3 mm in width located dorsal to the beak. The lesion was measuring up to 100 mm in length, 30 mm in height and 55 mm in width in total (Fig 1). There was destruction of the dorsal and lateral aspect of the casque at the rostral aspect of the lesion, which was more severe on the right side. The changes suggested the presence of an aggressive neoplastic process of the casque such as a squamous cell carcinoma. Other differential diagnoses such as inflammatory or infectious granulomas were not considered likely and other neoplastic processes were considered less likely. There was also thinning and interruption of the bone separation between the casque and the right nasal cavity at the right ventral aspect of the lesion. Mild thickening of the right dorsal nasal turbinate was observed at the right ventral aspect of the mass, indicative of mild inflammation or possible neoplastic infiltration. There were no signs that the mass had metastasized to other organs.

**Fig 2:**
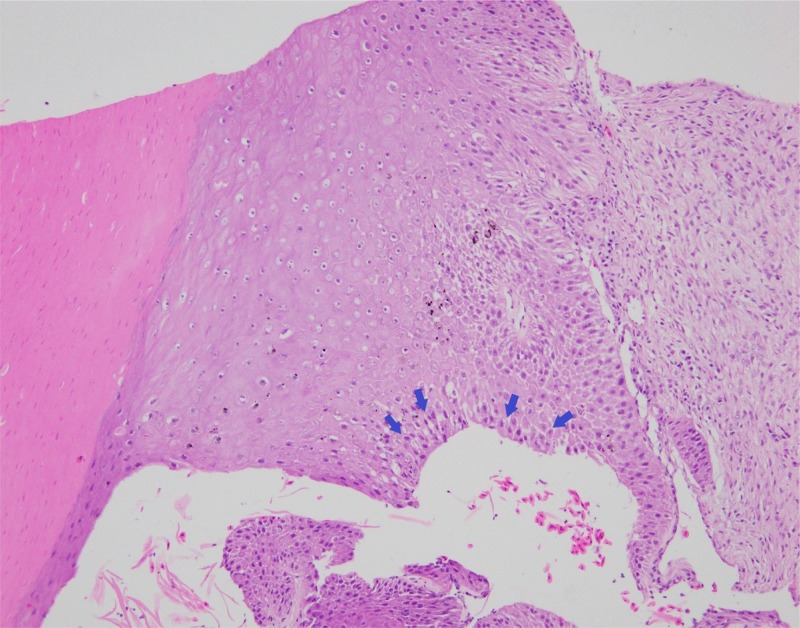
Histopathological examination revealed a thick layer of cornified squamous cells (arrows) with prominent nucleoli.

Three CT-guided biopsies of the mass were collected using a Tru-cut needle (Super-Core Biopsy Needle, Jorgensen Laboratories, Jorvet, 18ga x 90 mm) and submitted for histopathology. No haemorrhage was noted. These biopsies revealed a thick layer of irregular laminated keratin with necrotic cellular debris in the centre of the section. The base of the layer of squamous cells was 5 to 7 cells thick and the cells were plump with one to two prominent nucleoli (Fig 2). The cells showed moderate pleomorphism with mitotic figures about 1 to 4 per high power field. Squamous cell carcinoma was therefore suspected.

Flumazenil (Anexate, flumazenil 0.1 mg/ml, Hoffman-La Roche Ltd, Basel, Switzerland) 0.01 mg/kg IV was administered prior to recovery, which was unremarkable, and the patient was transported back to Jurong Bird Park avian hospital. Ceftiofur was continued for a total of eight doses, with the last dose administered 24 days after initial presentation.

As squamous cell carcinoma was strongly suspected based on the biopsy, the treatment options which would maintain a reproductively active bird included long-term non-steroidal anti-inflammatory drugs (NSAID) to reduce inflammatory cytokines and/or excision of the lesion with wide surgical margins. Given the location of the tumour, radical excision would expose a significant proportion of the casque space and maxillary sinus post-operatively [12]. Therefore, plans were made to have a 3D-printed casque prosthesis produced to be fitted after the excision, both for protection of the exposed structures and to allow the bird to express normal behaviours. An NSAID, meloxicam (Metacam, meloxicam 1.5mg/ml, Boehringer Ingelheim, Ingelheim/Rhein, Germany) was prescribed to be given once a day orally at 1 mg/kg, commencing 23 days after initial presentation and to be continued indefinitely.

### Creation of the 3D-printed casque replacement

The scanning data were exported in digital imaging and communications in medicine (DICOM) format and imported into medical imaging software InVesalius 3.0 for Windows (Renato Archer Information Technology Center, Campinas, Brazil) and Artec Studio software, version 9 for Windows (Artec 3D, Luxemburg) for 3D reconstruction and segmentation respectively. The 3D model of the dorsal maxillary beak and casque was isolated, and exported as STereoLithography (.stl) file for computer aided design (CAD) process.

All CAD was undertaken using Rhino software version 6 for Window (Rhinoceros, Robert McNeel & Associates, Seattle, WA). The main considerations for the surgical cutting guide design were to fit with the rhampotheca anatomy of the patient and delineate the planned surgical margins and guide the surgeon during the resection procedures.

The cutting guide needed to fit the anatomy of the dorsal maxillary beak and casque to ensure it could be fixed at the surgical site and clearly identify the planned surgical margins during surgery. The prosthesis was intended to cover the surgical wound after casque and beak resection.

Since the casque of the hornbill species may have an acoustic function [12], it was necessary for the shape of the artificial 3D-printed prosthesis to be identical in shape to the original beak and casque. This was assumed to be the best way to retain as many original acoustic properties as possible, and minimise any changes to the patient’s vocalisations. Placing the prosthesis accurately to overlap the planned surgical margins, would be necessary to seal the surgical wound, reducing the risk of contamination and infection in the post-operative period.

After the design process, the surgical cutting guide and prosthesis were produced by 3D printing. The EOS PA 2200, polyamide 12, was selected as an appropriate material to ensure a suitable physiological and biomechanical fit for the patient, and produce a robust prosthesis for long-term placement. EOS P396 3D printer (EOS GmbH Electro Optical Systems, Krailling, Germany) was used to produce the designed model. The duration of the printing process was approximately 12 hours.

**Fig 3:**
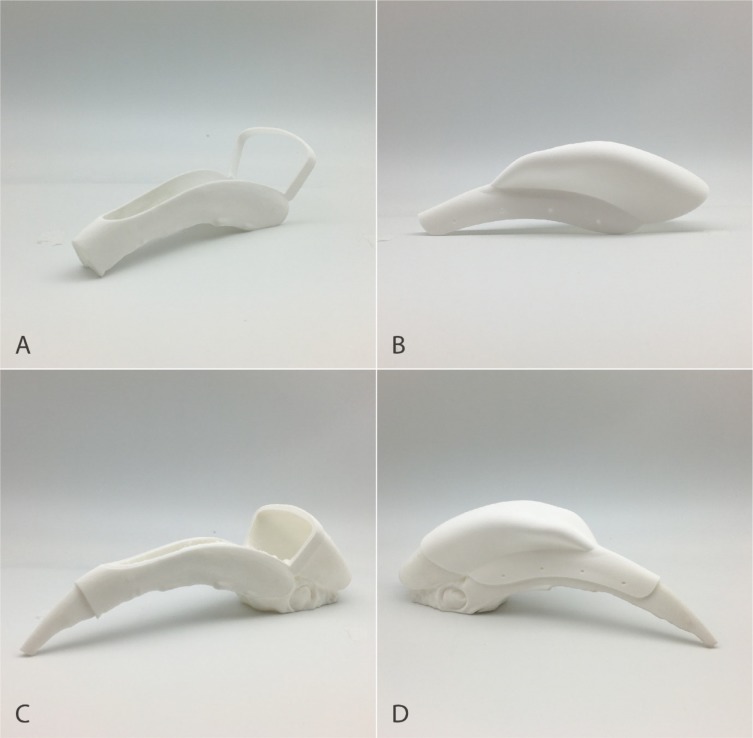
3D printed models. A: Surgical cutting guide; B: Prosthesis; C: Cutting guide on the testing model; D: Prosthesis on the testing model.

The final 3D printed surgical cutting guide and prosthesis are illustrated in Fig 3. The cutting guide was designed as a sliding structure, which could be slid along the beak and casque to overlie the surgical region (Fig 3C). Based on the properties of the polyamide 12 material, the prosthesis with 2mm thickness had sufficient hardness and a total weight of only 45g. To evaluate the fitting of the customized cutting guide and prosthesis, a replicated full dorsal maxillary beak and casque model which the area of the planned surgical margins was removed was 3D-printed. The testing demonstrated that the surgical cutting guide and the prosthesis matched the anatomy of the patient and the planned surgical margins were matched appropriately.

## Results

### Radical excision of the casque squamous cell carcinoma

The surgical procedure was planned to be performed 62 days after initial presentation of the patient. However, on the 60^th^ day post-presentation, a malodourous purulent discharge from the lesion developed, which required ceftiofur to be prescribed at the previous dose to decrease the chances of post-operative infection. Casque radiographs were also repeated on the same day under manual restraint and the rostral lesion noted to have increased in size and depth. However, no metastases were noted and the planned surgical margins were still appropriate despite the larger lesion. A repeat CBC and serum biochemistry was also performed, revealing a total leukocyte count that was mildly increased with monocytosis.

**Fig 4:**
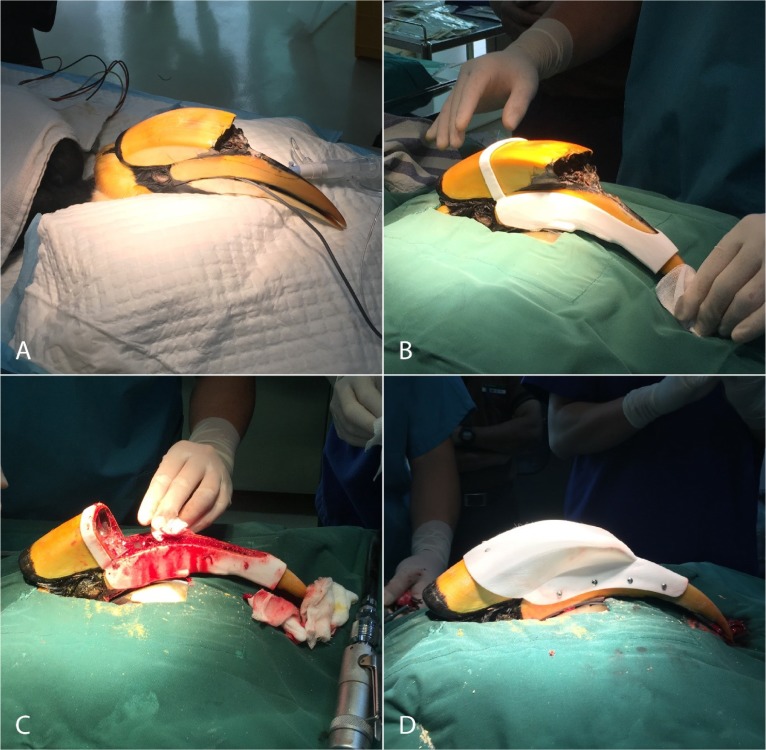
Casque/Beak resection and replacement using a 3D printed prosthesis. A: Anesthetised patient before receiving the surgery; B: The patient with the 3D printed surgical cutting guide; C: The tissue affected by carcinoma was removed; D: The 3D printed prosthesis was placed and secured using cortical screws to cover the surgical wound.

On the day of the surgery, food was withheld from the bird for 4 hours prior to surgery, and water for 2 hours. The bird was premedicated with intramuscular injections of 0.5 mg/kg midazolam and 0.5 mg/kg butorphanol, and transported to Singapore Zoological Gardens veterinary hospital. Induction and intubation were as previously described, and an appropriate surgical plane of anaesthesia was maintained at a concentration of 3–4% isofluorane throughout the surgery. A 22G IV catheter was placed in the right ulnar vein to permit intravenous administration of Hartmann’s solution at a rate of 10 ml/kg/h. Cloacal temperature was monitored periodically with a digital rectal thermometer, respiration rate and EtCO_2_ by in-line capnography, and heart rate and SpO_2_ via pulse oximetry. After a surgical field was disinfected with chlorhexidine, the 3D printed surgical cutting guide was slid into place on the hornbill’s maxillary beak and casque. Following the outline of the cutting guide, the carcinoma, together with a wide margin of normal rhinotheca and maxillary bone, was resected with an orthopaedic oscillating saw (DePuy-Synthes, Solothurn, Switzerland). After ensuring haemostasis, the 3D printed prosthesis was affixed to the maxillary beak using 2.7 mm cortical screws (DePut-Synthes, Solothurn, Switzerland) at intervals of 33 mm around the perimeter of the surgical margins. Dental acrylic was used to seal any remaining gaps between the prosthesis and the casque/beak. The duration of the surgery was 1.5 hrs; the surgical procedure is summarized in Fig 4.

**Fig 5:**
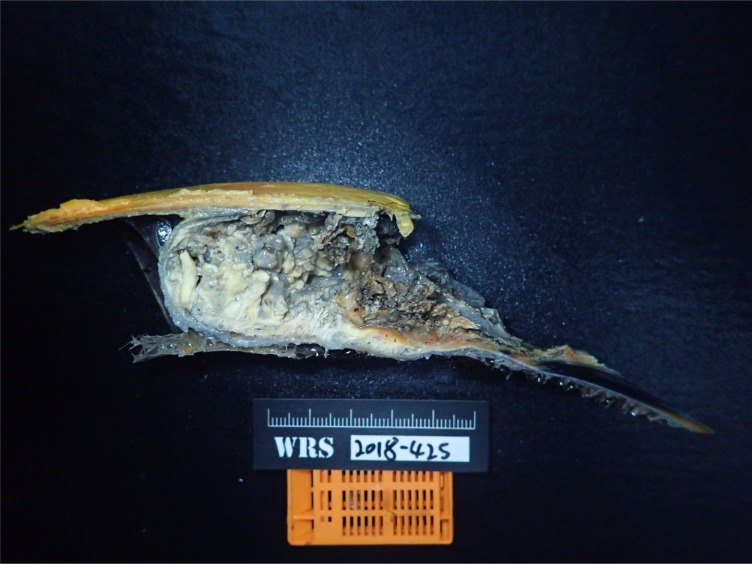
Sagittal section of the casque reveals abundant caseous materials in the cavity.

**Fig 6:**
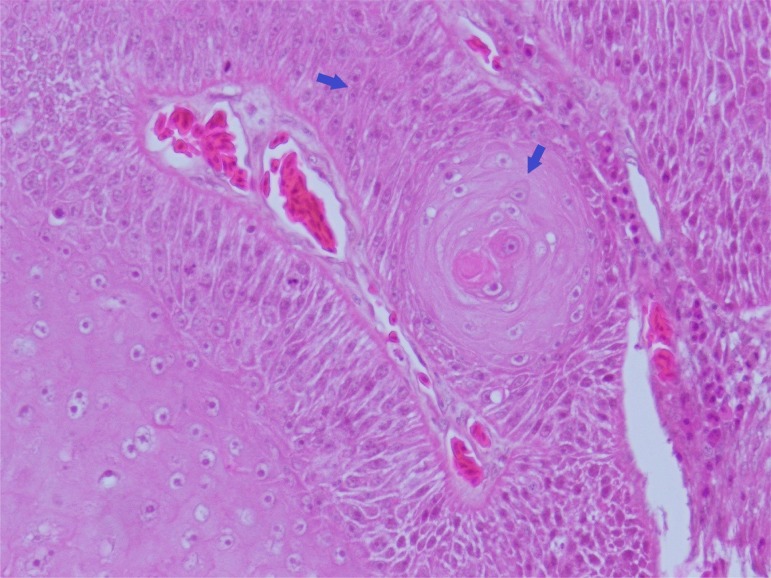
Microscopic examination of the tumor. The tumor is composed of large pleomorphic squamous cells (blue arrows) with abnormal cornification.

The removed casque was sent for histopathological examination. The casque was dissected sagittally. The cavity was filled with abundant caseous materials and foul-smelling necrotic tissue (Fig 5). The underlying bone was also removed by the surgery. There were clear margins throughout the resected tissue, albeit thin in the middle of the casque. Histopathologically, the section showed a layer of reactive bone tissue with overlying thick neoplastic squamous epithelial cells. The neoplastic cells presented marked pleomorphism. Abnormal cornification was also observed among the cellular layer. Toward the centre of the tumour, it was mainly composed of hyperparakeratotic tissue mixed with necrotic cellular debris and bacterial colonies. In the thinnest part of the surgical margin, the bone was still intact and the tumour cells was blocked by the bone tissue (Fig 6).

## Discussion

**Fig 7:**
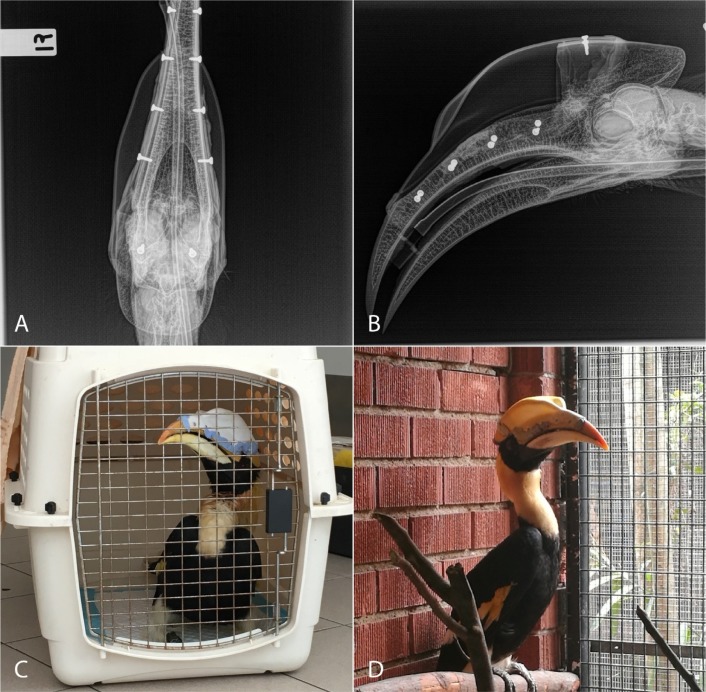
A: Dorsoventral view of the postoperative radiograph; B: Lateral view of the postoperative radiograph; C: The patient immediately after the surgery; D: The patient two months after the surgery.

The surgery was performed smoothly and without complications. Postoperative radiographs illustrated that the affected tissue was completely excised (Fig 7A and 7B). Recovery from anaesthetic was slow, likely due to the length of the procedure, but unremarkable. No flumazenil was administered post-operatively. For post-operative analgesia, tramadol (tramadol 50 mg/ml, Specialist Compounding Pharmacy, Singapore) was added to the treatment regime for additional analgesia at 30 mg/kg twice a day, administered in food.

Three days post-operatively, the bird appeared comfortable on a combination of tramadol and meloxicam, was eating normally and showed the same usual behaviours and activity as pre-operatively. He was manually restrained every three days for physical examination and monitoring of the prosthesis, and for intramuscular administration of ceftiofur. The dental acrylic applied at the edges of the prosthesis began to erode away early in the postoperative period, though the screws remained well-seated and there was no movement of the implants. A small amount of serosanguinous discharge was noted in the 48 hours after surgery, but by day three the bird had begun to show normal casque-colouring behaviours, and was seen rubbing the prosthesis across the uropygial gland to apply the yellow sebaceous secretions. Bloodwork performed four days post-operatively showed decreases in the total leukocyte count, and resolution of monocytosis. Serum biochemistry remained normal.

Tramadol was discontinued 7 days post-operatively, while meloxicam was continued twice a day for 10 days, and then decreased to once a day. Ceftiofur was continued post-operatively for 10 days to complete a total course of three doses. As the surgical site was unable to be visualised after prosthetic application, a choanal swab was taken five days post-operatively to screen for tracking infection. *Enterococcus faecalis* was isolated, sensitive to amoxycillin/clavulanic acid and doxycycline. As a mild malodour developed over the following days, doxycycline (Doxyvet Liquid, doxycycline 50 mg/ml, Vetafarm, Wagga Wagga, Australia) was commenced at 50mg/kg by intramuscular injection every 7 days for 14 days. The malodour was not associated with any discharge, weight loss or behaviour change. Bloodwork showed improvement following this course of antimicrobials, and the bird was bright, alert, normally responsive and showed normal food prehension and intake. All medications were discontinued, except for meloxicam.

31 days postoperatively, the bird was eating and drinking normally, showing normal behaviour and appeared to show no consternation regarding the prosthesis. However, watery diarrhoea developed and faecal cytology showed significant population of budding yeast and protozoan parasites. Antifungal and antiprotozoal therapy, administered in food, were commenced with itraconazole (Sporanox, itraconazole 10 mg/ml, Johnson & Johnson Pte Ltd, Singapore) at 5mg/kg once for 10 days and metronidazole (Metrogyl, metronidazole 50 mg/ml, J. B. Chemicals and Pharmaceuticals Ltd, Ankleshwar, India) 30mg/kg twice a day for 7 days respectively. The diarrhoea resolved and repeat faecal testing after 10 days showed no yeast, protozoa nor other endoparasites. These medications were therefore discontinued.

The bird was discharged from hospital to the exhibit 48 days after surgery. The patient’s CBC and serum biochemistry on discharge showed no abnormalities other than a mild monocytosis. Meloxicam was to be administered indefinitely at 1 mg/kg in a food item once daily. The patient’s discharge weight was 2.15kg, which had remained stable between 2.1 and 2.2kg throughout treatment and hospitalisation. The bird was discharged to an aviary in which he would be the sole occupant; the aviary had been renovated and replanted specifically to house him. A female conspecific was housed in an identical and adjoining enclosure next door, with a view to future pairing. Repeat radiographs and bloodwork up 90 days after surgery did not reveal any recurrence of the tumour nor any change in the patient’s overall health status.

The material used to create the prosthesis and surgical guide, the EOS PA 2200, polyamide 12, was selected based on the requirements of the case. Choosing biocompatible material was necessary to minimise tissue reaction from long-term contact with the prosthesis. The casque is a lightweight structure [12]. To increase the acceptance chance of the prosthesis by the bird, the customized prosthesis should not exceed the weight of the tissue resected. Since the bird would normally use the beak to manipulate items and his environment, the prosthesis was required to be tough enough to withstand the associated forces. The natural behaviour of the great hornbill was another important consideration. This species uses xanthous sebaceous secretions from the uropygial gland to colourise the keratin of the beak and casque. The aim of using a material which could take up these biological dyes was so that this behaviour would remain unaffected after surgery. Lastly, since there is a clear link between UV radiation exposure and the development of basal cell carcinoma in humans [15], it is possible that UV exposure is a factor in the etiology of squamous cell carcinoma in great hornbills [16]. Therefore, ideally the material used in the printing of the prosthesis would provide a measure of UV protection.

Observation of this bird in his usual captive environment suggests that there is complete acceptance of the 3D printed prosthesis as part of its own body. This is evident from hornbill’s displaying natural colouration behaviour, which coupled with the ability of the material used to take up biological pigments, enabled the prosthetic casque to appear similar in colour and texture to the original rhinotheca. Preliminary observations show that the prosthesis can act as a resonance chamber (a major function of the natural casque), though further audio analysis is required to evaluate its efficacy in this function. Due to the novelty of this procedure, it is not known how healing at the surgical margins will progress. Granulation and remodelling at the surgical margins may form a permanent adhesion to the prosthesis or remodelling may progress in the shape of the original structure.

Based on the outcome of this case, medical imaging and 3D printing can be considered a useful approach in the design and production of customized surgical cutting guides and prostheses in veterinary surgery. Collaboration between designers and veterinarians throughout the design process can result in a customized prosthesis which permits natural behaviours with good acceptance.
